# (Re)examining the Relationship Between Children’s Subjective Wellbeing and Their Perceptions of Participation Rights

**DOI:** 10.1007/s12187-016-9396-9

**Published:** 2016-05-23

**Authors:** Katrina Lloyd, Lesley Emerson

**Affiliations:** School of Education, Queen’s University Belfast, University Road, Belfast, BT7 1NN UK

**Keywords:** Wellbeing, Children’s rights, Participation, Survey

## Abstract

In recent years wellbeing has been linked increasingly with children’s rights, often characterised as central to their realisation. Indeed it has been suggested that the two concepts are so intertwined that their pairing has become something of a mantra in the literature on childhood. This paper seeks to explore the nature of the relationship between wellbeing and participation rights, using a recently developed ‘rights-based’ measure of children’s participation in school and community, the Children’s Participation Rights Questionnaire (CPRQ), and an established measure of subjective wellbeing – KIDSCREEN-10. The data for the study came from the Kids’ Life and Times (KLT) which is an annual online survey of Primary 7 children carried out in Northern Ireland. In 2013 approximately 3800 children (51 % girls; 49 % boys) from 212 schools participated in KLT. The findings showed a statistically significant positive correlation between children’s overall scores on the KIDSCREEN-10 subjective wellbeing measure and their perceptions that their participation rights are respected in school and community settings. Further, the results indicated that it is the social relations/autonomy questions on KIDSCREEN-10 which are most strongly related to children’s perceptions that their participation rights are respected. Exploration of the findings by gender showed that there were no significant differences in overall wellbeing; however girls had higher scores than boys on the social relations/autonomy domain of KIDSCREEN-10. Girls were also more positive than boys about their participation in school and community. In light of the findings from this study, it is suggested that what lies at the heart of the relationship between child wellbeing and children’s participation rights is the social/relational aspects of both participation and wellbeing.

## Introduction

In recent years, the concept of children’s quality of life and wellbeing has become firmly embedded in discourse and policy in the academic, government and public sectors in countries across the world (Camfield et al. 2009; Morrow and Mayall 2009; Ereaut and Whiting 2008; Statham and Chase 2010). Child wellbeing is important not only for future outcomes for children as adult members of society, or their ‘well-becoming’ (Ben-Arieh 2008 p6), but also for their present lives in what has been termed ‘a good childhood’ by The Children’s Society (2009 p2). Morrow and Mayall (2009) and Fattore et al. (2009) also draw attention to the importance of monitoring childhood wellbeing in the ‘here-and-now’ but suggest that this should be undertaken using an approach that recognises and encompasses children’s rights. This accords with the views of other scholars in the field; indeed in recent years wellbeing has been linked increasingly with children’s rights, often characterised as central to their realisation (Kennan et al. 2011; Bradshaw et al. 2007). The link is unsurprising given that those concerned with child wellbeing and children’s rights are essentially concerned with seeking improvement in children’s lives (Lundy 2014). Moreover, children’s rights have, like wellbeing, become an important part of policy and academic discourse (Henricson and Bainham 2005; Reynaert et al. 2009), particularly since the ratification of the United Nations Convention on the Rights of the Child (CRC) (United Nations, UN 1989) by most countries in the world. As such the CRC has become described variously as ‘the dominant image of contemporary childhood’ (Kellett et al. 2004, p34) and a ‘backdrop’ to childhood research and policy discourse (McKechnie and Hobbs 2004, p282).

However, while Lundy (2014, p84) notes that the two concepts – children’s rights and wellbeing – are so intertwined that their pairing ‘has become something of a mantra’ in the literature on childhood, she argues that the concepts are ‘nonetheless distinct’ in terms of rationale and scope. First, children’s rights standards reflect the bare minimum conditions to which states have agreed to be held to account (see also, Tomasevski 2003), which arguably fall short of the common definitions of child wellbeing. Secondly, children’s rights discourse positions children as *entitled* to (rather than *needing*) protection and suitable provision and *entitled* to participate in decisions affecting their own lives. Thirdly, a comparison between the CRC and the common domains of wellbeing, Lundy (2014) suggests, indicates that the substantive CRC rights, though limited to issues within the state’s capacity, are broader in scope, particularly in relation to children’s civil and political rights (right to information, freedom of expression, assembly and conscience).

Nonetheless, despite the conceptual tensions between ‘child wellbeing’ and ‘children’s rights’ an emerging body of empirical evidence is being drawn upon in academic discourse to suggest a link between the two, notably in relation to children’s *participation* rights. For example, research evidence from a large scale survey of young people in Spain reported a relationship between children’s subjective wellbeing and their knowledge of the CRC, and between their subjective wellbeing and perceptions of the extent to which they can participate in decisions made in school and community settings (UNICEF Spain 2012; Casas et al. 2013). Moreover, analyses carried out on the same data by González et al. (2015) found a statistically significant, positive relationship between subjective wellbeing and children’s perceptions of their participation in decision-making taken at home. These findings resonate with results from surveys conducted with young people in Northern Ireland which indicate that their subjective wellbeing is related to a belief that they are involved in making decisions about their lives (ARK 2010; Lloyd and Schubotz 2014).

However, while the items used in these surveys to measure decision-making and participation draw on key ideas located in the literature on children’s participation in general (such as having views sought and feeling listened to), it could be suggested that they do not address fully what would be considered as a comprehensive ‘child rights-based’ measure of participation. This paper seeks to explore further the relationship between children’s participation rights and their subjective wellbeing, through a discussion of the findings from a large-scale survey of children attending schools in Northern Ireland. The survey included, inter alia, a recently developed ‘rights-based’ measure of children’s participation in school and community, the Children’s Participation Rights Questionnaire (CPRQ) (Emerson and Lloyd 2014), and an established measure of children’s subjective wellbeing, KIDSCREEN-10 (The KIDSCREEN Group Europe 2006).

The paper begins with a brief discussion of perspectives on wellbeing and children’s participation rights by way of justifying the constructs used in this study. Extant evidence pertaining to the relationship between children’s subjective wellbeing and children’s participation in decision making is then reviewed, identifying a need to explore further how participation rights, as articulated in the CRC, relate to wellbeing. Following a discussion of the methods used in this study, statistical analyses and discussion of the findings are presented. The paper concludes by suggesting that the findings from this study add significantly to the empirical evidence base for a positive correlation between child wellbeing and children’s participation rights. Moreover, it is suggested that the key to understanding this relationship lies arguably in the social or relational aspects of participation, in particular the ways in which children’s perceptions of social relations/autonomy and adult support for their participation intersect.

### Defining Wellbeing and Children’s Participation Rights

#### Wellbeing

There is no universally agreed definition of wellbeing and the term is often used interchangeably with ‘life satisfaction’, ‘happiness’ and ‘quality of life’ (Statham and Chase 2010; Selwyn and Riley 2015). What is agreed, however, is that wellbeing is multifaceted (Delle Fave et al. 2011; Forgeard et al. 2011; Dodge et al. 2012) and encompasses both objective (e.g. income, education, health) and subjective (e.g. inter-personal relationships, autonomy) aspects of a person’s life (Bowling 2011; Casas 2011; Forgeard et al. 2011; Selwyn and Riley 2015; Statham and Chase 2010). According to Casas (2011, p 564), while the emphasis within the conceptualisation and measurement of wellbeing has mostly involved adults, ‘the satisfaction of children and adolescents continues to be treated as irrelevant’. Ben-Arieh (2008), too, drew attention to the importance of assessing and monitoring child wellbeing and of creating measures that encompass the multi-dimensional aspects of children’s lives within the framework of Bronfenbrenner’s (1992) ecological systems theory.

Bronfenbrenner’s model emphasises the need to consider children’s development within the context of the interrelations between their own characteristics and the environment in which they live including family, friends, school and the wider community (Bronfenbrenner 1992; Ben-Arieh 2008; Scott et al. 2015). However, while acknowledging the usefulness of the ecological model in understanding child wellbeing, Ben-Arieh (2007) suggests that what is lacking is the children’s own perspective and experience. As Casas (2011, p559) notes, ‘the point of view of the youngest generations is also important to understand our societies, particularly those aspects of social life involving or affecting them’. Furthermore, assessing wellbeing from children’s own perspective is necessary because they have been shown to differ from adults in some of the ways in which they define wellbeing and the aspects they prioritise (Fattore et al. 2007, 2009; Sixsmith et al. 2007; Statham and Chase 2010). For example, both adults and children included family, home and friends in their conceptualisation of children’s wellbeing in a study carried out by Sixsmith et al. (2007). However, while adults tend to prioritise what Fattore et al. (2009, p70) term ‘traditional domains derived from protection or service-oriented rationales’ such as safety and economic wellbeing, children emphasise a positive sense of self, agency and feelings of security (Fattore et al. 2009).

Cognisant of the similarities and differences in adults’ and children’s conceptualisations of wellbeing, and of the necessity of moving away from regarding children as ‘unreliable respondents’, Ravens-Sieberer et al. (2005 p354) developed KIDSCREEN. The KIDSCREEN has three versions – 52, 27 and 10 – and is a subjective health-related quality of life questionnaire that encompasses dimensions of wellbeing identified as important by both adults and children including physical and psychological health, relationships with parents and friends, and autonomy. This study uses the KIDSCREEN-10 to measure wellbeing and the paper will explore the extent to which the overall measure, and the domains assessed within it, relates to children’s perceptions of their participation rights.

#### Wellbeing and Gender

In general, most studies report that the majority of children and young people have high levels of overall subjective wellbeing (Dinisman and Ben-Arieh 2016; The Children’s Society 2014; Unicef Spain 2012); however, the research findings in relation to gender are mixed. Some studies have reported differences (Rees and Dinisman 2015; Unicef Spain 2012) while others have not (e.g. Huebner et al. 2006). According to Dinisman and Ben-Arieh (2016), these inconsistent findings may be due in part to the measures of subjective wellbeing used. They contend that while there appear to be no gender differences in general overall life satisfaction measures, boys tend to have higher scores on domains such as appearance and self-image, for example, while girls have higher scores on school and interpersonal relationships. Support for this proposition comes from studies using the multi-dimensional KIDSCREEN-27 measure of subjective wellbeing; boys have better wellbeing on two domains (physical health and psychological well-being) while girls report better wellbeing on three domains – school, autonomy/parents and social support and peers (KIDSCREEN Group Europe 2006; Lloyd 2011; Ravens-Sieberer et al. 2007). In contrast, studies using the shortened KIDSCREEN-10 have reported inconsistent findings with some reporting no significant gender differences (e.g. Lloyd 2013) and others suggesting that boys have higher wellbeing than girls using this overall measure (e.g. Erhart et al. 2009; Ravens-Sieberer et al. 2010). Given the mixed results in relation to gender differences in the KIDSCREEN-10, this will be explored in the present study.

#### Children’s Participation Rights

While there is a lack of agreement on what constitutes the rationale for, and scope of, children’s participation (Herbots and Put 2015; Thomas 2007) discourse surrounding it centres largely on challenging the dominance of adult-centric agendas and structures (Wyness 2012; Thomas 2007) and the resultant tokenistic nature of children’s involvement in decision making (Hart 1997). This challenge usually draws attention to children’s competence and agency (James et al. 1998; John 2003) or stresses the importance of recognising the political status of children (Lockyer 2003) as citizens who possess democratic rights (Holden and Clough 1998; Matthews 2003; Howe and Covell 2005). In relation to the latter in particular, the influence of the CRC has undoubtedly resulted in children’s participation becoming somewhat synonymous with children’s rights (Lansdown 2009).

The right of children to have their views (or ‘voice’) given due weight in all decisions that affect them, enshrined in Article 12 of the CRC, is often regarded as the primary provision of the CRC in relation to children’s participation rights. However, as is the case for all human rights provisions, which are characterised as indivisible and interrelated (Steiner and Alston 2000), Article 12 cannot be seen in isolation (Lundy 2007). Rather, as Skelton (2007 p.167) notes ‘participation is not a stand-alone article but is embedded within the CRC’. Thus, a ‘rights-based’ approach to children’s participation must first take account of other provisions within the CRC and its associated jurisprudence. As Article 12 states, those acting on behalf of the state should ‘assure to the child who is capable of forming his or her own views the right to express those views freely in all matters affecting the child, the views of the child being given due weight in accordance with the age and maturity of the child’ (UN 1989).

The Committee on the Rights of the Child, who monitor how states are complying with the CRC, has explained that such rights-based participation should thus be voluntary (that is, a right and not a duty of the child), respectful, inclusive, safe and in particular facilitated by child-friendly approaches to actively soliciting children’s views (UN 2009). The Committee also recognises that the child’s right to be heard (UN 1989, Article 12) is closely aligned to his or her right to information (UN 1989, Article 17) which they describe as ‘a prerequisite for the effective realization of the right to express views’ (UN 2009 para. 82). Further they state that the child’s right to freedom of expression (UN 1989, Article 13) ‘relates to the right to hold and express opinions, and to seek and receive information through any media’ (UN 2009 para.81). Importantly, a ‘rights-based’ approach to participation takes into account the adult-child power dynamic. Article 5 (CRC) recognises that children will on occasion need support in the exercise of their rights in accordance with their evolving capacities. As such, adults are positioned in the CRC as ‘enablers’ capable of playing a positive role in guiding and assisting children in the formation and expression of their views.

The extent to which children’s views are sought on matters that affect them is thus only one component of the child’s right to participate. What is more significant is the way in which these views are sought, how children’s autonomy is balanced with support from adults in expressing their views and the extent to which their views are taken seriously and acted upon. This ‘rights-based’ construct of participation is the lens through which this study explores the relationship between children’s wellbeing and participation rights.

#### Participation and Gender

According to González et al. (2015) ‘very few studies have explored participation from a gender perspective’ (p 96). To address this, González and colleagues analysed data from a survey of 6000 young people in Spain and found that girls reported more participation in the family context than boys. González et al. (2015), drawing on research by Navarro (2011) also note that participation is generally perceived by young people as being more typical of girls than boys (cited in González et al. 2015, p 96). Other research, too, has reported gender differences in attitudes to, and participation in, school and community contexts among young people. More girls than boys believe that participating in school is valuable and the former are also more likely to be involved in school councils (Nelson et al. 2010) and to volunteer in the community (Albanesi et al. 2015; Irvine and Osborne 2014; Wilson 2000) than the latter. Similar findings were reported by children taking part in a large-scale survey in Northern Ireland; more children said they were asked for their opinion in school (58 %) than at home (41 %) and girls were more likely than boys to say they had been asked for their opinions in both contexts (ARK 2010). This paper will explore gender differences in participation in the context of school and community using a questionnaire specifically designed by children to capture this concept from their perspective.

Having outlined the theoretical perspectives on child wellbeing and children’s participation rights which underpin this paper, attention now turns to the extant evidence relating to the relationship between these two concepts.

#### Exploring the Relationship Between Children’s Participation Rights and Wellbeing

Much of the research into child wellbeing from a children’s rights perspective has focused on ‘objective’ measures such as the extent to which children and young people have access to childcare, education and employment; this allows for comparisons across countries and over time (Bradshaw et al. 2007; UNICEF 2007). Less common are studies that examine the relationship between children’s participation and wellbeing from their own perspective; yet, as noted at the outset of this paper, those that do have reported a clear relationship between them.

For example, exploring the involvement of children in decision making projects at school and in the community in six countries across Europe, Kränzl-Nagl and Zartler (2010) reported an enhanced sense of importance and self-esteem among those who had participated. Further, in 2012, a survey carried out in Spain asked approximately 6000 young people in their first year of compulsory secondary education about their perceived participation in decision making at home, school and in their community. While the percentages of children who felt they were involved in decision making in these three domains of their lives were generally low, those who reported higher levels of perceived participation had better subjective wellbeing (Casas et al. 2013; UNICEF Spain 2012). Also using these data from Spain, but focusing specifically on participation within the context of the family, González et al. (2015) reported that adolescents who perceive they have more participation at home have better subjective wellbeing than their peers who do not. In addition, these authors report mixed results in relation to gender; girls who perceive they participate at home had higher wellbeing scores on a measure of satisfaction with a range of life domains including school, health and relations and a Student Life Satisfaction scale. In contrast, boys who felt they participated at home had higher scores than girls on a single item overall life satisfaction measure (González et al. (2015).

Similar findings have been reported from surveys of children and young people in Northern Ireland, with better subjective wellbeing reported among those who felt they were involved in decision making at home and in school (Schubotz et al. 2007; Lloyd and Schubotz 2014). In 2010, over 5000 children aged 10 and 11 years from across Northern Ireland were questioned in a survey about whether they had been asked by a teacher for an opinion about the way that something was run in their school. They were also asked whether their parents or guardians usually asked for their opinion when making major decisions about things happening at home that might affect them. In contrast to the findings reported by González et al. (2015), there were no gender differences; both boys and girls who said they had been involved in decision-making at home and school had significantly higher scores, and therefore better subjective wellbeing, than their peers who had not been involved (ARK 2010).

Taken together, the findings from these studies suggest that involving children and young people meaningfully in decisions that affect them may confer real benefits to their wellbeing. Therefore one aim of the current research is to add to this emerging empirical evidence by exploring whether there is a statistically significant relationship between children’s wellbeing and their perceptions of participation rights in school and community. However, the items used in previous surveys in relation to ‘participation’ arguably do not address fully what would be considered as a comprehensive ‘children’s rights-based’ measure of participation. First, the items tend to focus on the extent to which children feel ‘listened to’ or ‘can participate in decisions’ (UNICEF Spain 2012) or are ‘asked for an opinion’ (ARK 2010). As discussed above, this attends primarily to only one component of the child’s right to participate: having their views sought. What is missing from these measurements is the extent to which children feel they are supported by adults in expressing their views and the extent to which these views are taken seriously and acted upon. Secondly, previous research studies exploring children’s participation and wellbeing, particularly in relation to school and community, have tended to rely on individual questions developed mostly by adults, which may not accurately reflect what participation in these contexts means from the child’s perspective. A rights-based approach suggests that children should be involved in the development of such questions (Lundy and McEvoy (Emerson) 2012a, b).

To address these limitations, this study used a recently developed children’s participation rights questionnaire (CPRQ) (Emerson and Lloyd 2014). The measure was developed in conjunction with a group of child co-researchers, drawing on an established children’s rights-based approach to research (Lundy and McEvoy (Emerson) 2012a, b). One feature of this approach is to build children’s capacity in understanding the key concepts associated with the research study – in this case children’s participation rights. The resultant measure was thus conceptually sound in relation to the articulation of participation from the perspective of the CRC but, importantly, reflected the perspectives and lived experiences of children themselves, with the items presented in an authentic language children would use (for more details on the process see Emerson and Lloyd 2014). Based on previous empirical research, the main hypothesis proposed in this study is that there will be a statistically significant correlation between children’s perceptions of their participation rights and overall wellbeing using KIDSCREEN-10. Further, given that both questionnaires include items assessing children’s perceptions of autonomy and adult support, it was hypothesised that this would explain the significant relationship between them.

## Method

The data for this study came from the 2013 Kids’ Life and Times (KLT) which is an annual online survey of Primary 7 (P7) children carried out in primary (elementary) schools across Northern Ireland (ARK 2013). KLT began in 2008 and is run by ARK which is a joint initiative between Queen’s University Belfast and the University of Ulster (www.ark.ac.uk). Given that every P7 child in Northern Ireland has access to the Internet in school, and all schools are invited to participate in the survey, KLT is a representative sample of the views of all P7 children in Northern Ireland (Lloyd and Devine 2010).

### Participants

P7 children are aged 10 or 11 years and are in the final year of their primary school education; the majority will move to secondary school at this stage. In 2013 approximately 3800 children (51 % girls; 49 % boys) from 212 primary schools participated in KLT. The method for the survey – online in school – was developed in conjunction with children, parents and school principals following consultations carried out in 2008 (Lloyd and Devine 2010).

#### KLT Questionnaire

Each year, the survey consists of around 80 (mainly) closed questions and the children click on the answer that best applies to them. Core questions, including sex, long-term illness, family socio-economic characteristics and subjective wellbeing, are asked in each year of the survey while modules of questions, such as bullying at school, children’s rights and educational aspirations, are purchased by funders within particular survey years. In 2013, a measure of children’s rights participation was included in the survey, funded by Improving Children’s Lives which is based in Queen’s University Belfast. This paper, therefore, uses data from the two modules to examine the relationship between children’s participation rights and wellbeing among KLT respondents in 2013. There were several reasons for including the children’s participation rights questionnaire in KLT. First, the survey is methodologically robust and the findings can be generalised to all P7 children across Northern Ireland. Secondly, KLT includes a measure of subjective wellbeing every year and, finally, the age group of respondents – 10 and 11 years – is similar to the age of the children participating in the development of the children’s rights questionnaire.

### Instruments

The CPRQ (Emerson and Lloyd 2014) was developed in conjunction with child co-researchers and consists of 14 statements measuring children’s participation in school and community. Each statement has the responses ‘Never, Seldom, Quite Often, Very Often, Always’. The statements are consistent with the features of rights-based participation discussed above, for example: ‘I can give my opinions freely’; ‘The adults make it easy for me to give my views’; ‘The adults take my views seriously’; ‘The adults make sure I can easily get the information I need about what is going on’. Scores on the CPRQ range from 14 to 70 with higher scores representing more positive feelings about children’s enjoyment of their participation rights. The internal consistency of the CPRQ in this study, measured using Cronbach’s alpha, was .89 demonstrating that the questionnaire had good reliability (.70 is conventionally used as the threshold).

In the 2013 KLT survey, children’s wellbeing was assessed using the KIDSCREEN-10 instrument which is a short version of the KIDSCREEN-27 (Ravens-Sieberer et al. 2007) and is consistent with the construct of wellbeing discussed in this paper. The psychometric properties of the KIDSCREEN used in an on-line survey of P7 children in Northern Ireland have been shown to be robust (Lloyd 2011). The KIDSCREEN-10 measures health-related quality of life (HRQoL) from the child’s perspective and is designed for use with children aged between 8 and 18 years (The KIDSCREEN Group Europe 2006). The KIDSCREEN-10 is a valid measure of a general HRQoL factor in children and adolescents (Ravens-Sieberer et al. 2010) and has been shown to function as a good indicator of wellbeing. The children are asked to respond to the questions in relation to the past week and each has a 5-point response scale which is either ‘Not at all, Slightly, Moderately, Very, Extremely’ **or** ‘Always, Very often, Quite often, Seldom, Never’ depending on the wording of the question. Examples of the questions are: ‘Have you felt lonely?’; Have you been able to do the things that you want to do in your free time?’ ‘Have you got on well at school?’ Higher mean scores on the KIDSCREEN-10 indicate better wellbeing. The KIDSCREEN-10 data were analysed using the syntax files supplied on the compact disk that accompanied the KIDSCREEN handbook, and the scores that are presented in this paper are the international T-values (Mean = 100; SD = 50) (The KIDSCREEN Group Europe 2006). The internal consistency of the KIDSCREEN-10 for this sample of children, measured using Cronbach’s alpha, was .77 which indicates the questionnaire has good reliability.

### Procedure

Each year, all primary schools in Northern Ireland are sent letters, parental consent forms, teacher instructions and bookmarks for every P7 pupil in the weeks prior to the KLT survey going live on the internet – usually in the final term of the school year (April to June). Schools are given a unique identification number so that entries can be associated with a particular school; in this way, principals can be sent an anonymised report for their school to use for monitoring pupil attitudes towards issues such as bullying, happiness at school etc. The children are not asked for their names so it is not possible to identify individual pupils; only a school identification number is required to log on to the survey. Respondents complete the online questionnaire on computers in their classroom or in their school’s computer room. Each question has a skip option which the respondents can use if they do not want to answer a question. The survey takes about 20 min to complete.

### Ethics

Initial approval was given for the KLT survey to be developed and run in 2008 by the Ethics Committee in the School of Sociology, Social Policy and Social Work at Queen’s University Belfast. Subsequently, as the method has remained the same, the Ethics Committee is asked for approval of the questionnaire that will be used in each survey year and, to date, permission has been granted. Children can only take part in the survey if their school principals and their parents/guardians give their consent. At the start of the survey each year, the children are asked if they want to participate in KLT and, if not, they do not have to. Therefore, the children who take part in the survey are those for whom permission has been obtained from school, parent/guardian and pupil.

### Data Analysis

Descriptive statistics in the form of mean scores and standard deviations are presented for the variables of interest. T-tests are used to investigate gender differences for normally distributed scale variables (KIDSCREEN-10 and CPRQ) and Mann-Whitney U for non-normally distributed variables (KIDSCREEN-10 domains). Correlational analyses (Pearson’s and Spearman’s rho) are used to explore the relationship between children’s wellbeing and their participation rights and the Fisher z transformation to test the difference between correlation coefficients for boys and girls.

## Results

### Subjective Wellbeing

The mean KIDSCREEN-10 score was 52.57 (SD = 10.38). This was higher than the mean score for 11 year olds reported by Erhart et al. (2009) in 15 European countries^1^ taking part in the Health Behaviour in School-aged Children study (HBSC) (Mean = 50.67, SD = 10.71). However, the difference between the mean scores of the two groups of children was small as measured by Cohen’s d effect size (0.18).^2^ The HBSC study reported a statistically significantly higher mean KIDSCREEN-10 score for boys; in contrast, girls taking part in the 2013 KLT had a marginally higher mean KIDSCREEN-10 score than boys (Table 1) although the difference was not statistically significant (*t* = 1.82, df = 3728, *p* > 0.05).

**Table 1:** Mean subjective wellbeing scores

	Mean	SD
All children	52.57	10.38
Gender		
Boys	52.25	10.20
Girls	52.87	10.54

Given that previous research has reported mixed results in relation to gender differences within specific domains of the KIDSCREEN questionnaires (Lloyd 2011; The KIDSCREEN Group Europe 2006) this was explored in the present study. The KIDSCREEN-10 was recoded to reflect three dimensions of wellbeing – physical wellbeing (i.e. fitness, energy); psychological wellbeing (i.e. sad, lonely), and social relations/autonomy (i.e. time for self, relationships with parents and friends, school). As Table 2 shows, boys tended to have slightly higher scores on the physical (Mann-Whitney *U* = 1718246.50, *Z* = 1.11, *p* > 0.05) and psychological (Mann-Whitney *U* = 1,703,573, *Z* = 1.44, *p* > 0.05) domains of wellbeing than girls, although the differences were not statistically significant. In contrast, girls had statistically significantly higher mean rank scores than boys on the social relations/autonomy domain (Mann-Whitney *U* = 1,564,527, *Z* = 4.07, *p* < 0.001) although the strength of the relationship was weak (*r* = 0.07).

**Table 2:** Correlations between children’s participation rights and KIDSCREEN dimensions

	Mean rank	
KIDSCREEN dimensions	Boys	Girls
Physical wellbeing	1894.31	1855.90
Psychological wellbeing	1897.44	1847.97
Autonomy/Relationships	1769.44	1911.66

### Children’s Perceptions of Their Participation Rights

The mean score on the CPRQ for all children was 45.39 (SD = 11.20) and girls had a higher mean score than boys (Table 3). The difference was statistically significant (*t* = 9.38, df = 3549, *p* < 0.001). The girls who responded to KLT were, therefore, more positive about their participation rights in school and community than boys although the effect size, measured using Cohen’s *d*, was fairly moderate (*d* = .32).

**Table 3:** Mean CPRQ scores

	Mean	SD
All children	45.39	11.20
Gender		
Boys	43.57	11.03
Girls	47.06	11.09

### Overall Wellbeing and Children’s Participation Rights

The KLT data were analysed to examine the relationship between wellbeing and children’s participation rights among this sample of P7 children in Northern Ireland. Based on the findings reported by Unicef Spain (2012), it was expected that there would be a positive correlation between the CPRQ and the KIDSCREEN-10. As both variables approximated the normal distribution, Pearson’s correlation coefficient was calculated. The results showed a statistically significant positive correlation between the CPRQ and the KIDSCREEN-10 (*r* = 0.38, *n* = 3533, *p* < 0.001). Therefore, better overall subjective wellbeing was statistically significantly related to more positive feelings about participation rights in school and community. The two variables share around 14 % of their variance in common. Two separate correlations were carried out – one for girls and one for boys. The correlation coefficient between the two variables was higher for girls than for boys (.40 and .35 respectively) although the difference was not statistically significant (*z* = 1.64, *p* > 0.05).

### Exploring Domains of Wellbeing and Children’s Participation Rights

The findings reported thus far have supported previous research and demonstrated a statistically significant relationship between children’s participation in school and community measured using the CPRQ and overall wellbeing as assessed by the KIDSCREEN-10. Beyond that, one of the key aims of this study was to (re)examine children’s perceptions of participation rights and wellbeing in an attempt to elucidate what lies at the heart of this statistically significant relationship. To this end, correlations were carried out between the CPRQ and the three domains of wellbeing outlined in Section 3.1 above, namely physical wellbeing, psychological wellbeing, and social relations/autonomy. Given the potential intersection of items on the social relations/autonomy domain of KIDSCREEN-10 and the CPRQ, we speculated that it might be this aspect of wellbeing that could best explain the statistically significant correlation between the two concepts. The results from the correlations between these three dimensions of wellbeing with the CPRQ showed that although all three were statistically significant, the strongest relationship was between the social relations/autonomy questions on KIDSCREEN-10 and children’s participation rights for both boys and girls (Table 4). Furthermore, the difference between the correlations for boys and girls on this domain was statistically significant (.38 and .43 respectively; *z* = 1.96, *p* < 0.05). The implications of these findings for furthering our understanding of the relationship between children’s wellbeing and participation rights, and the limitations of the study, are discussed in the following sections.

**Table 4:** Correlations between children’s participation rights and KIDSCREEN dimensions

	KIDSCREEN dimensions								
	Physical wellbeing			Psychological			Autonomy/Relationships*		
	All	Boys	Girls	All	Boys	Girls	All	Boys	Girls
CPRQ	.25	.23	.27	.15	.15	.17	.41	.38	.43

*p < 0.05

## Discussion

Overall, these findings support the main hypothesis proposed and indicate that there is a statistically significant positive correlation between children’s subjective wellbeing and their perceptions that their participation rights are respected in school and community settings. The findings offer support for previous studies which found that children who reported higher levels of perceived participation had better subjective wellbeing (Casas et al. 2013; UNICEF Spain 2012). Further, whilst this study did not measure children’s enjoyment of their participation rights in the context of home, the findings resonate with the analyses carried out by González et al. (2015) and ARK (2010) which found a similar, statistically significant, positive relationship between children’s perceptions that they participate in decisions taken within the home and family and their subjective wellbeing. However, what is notable about the findings in this study is that the measure of participation used was designed deliberately to encompass *all* aspects of a children’s rights-based perspective on participation. These findings thus strengthen considerably the emerging empirical evidence suggesting a positive relationship between child wellbeing and children’s enjoyment of their participation rights.

In relation to gender, while there were no statistically significant differences in overall wellbeing scores (as measured by KIDSCREEN-10), there were significant differences between the social relations/autonomy domain scores of girls and boys, with the former having higher scores than the latter. These results support the findings from previous research which reported no gender differences in overall wellbeing using KIDSCREEN-10 among primary school-age children (Lloyd 2013). Furthermore the finding that boys had slightly better physical and psychological wellbeing than girls while the latter reported better wellbeing in the social relations/autonomy resonates with previous research using the five domains from KIDSCREEN-27 (KIDSCREEN Group Europe 2006; Lloyd 2011; Ravens-Sieberer et al. 2007). While these general trends support previous research, the failure of the analysis for this paper to detect statistically significant gender differences between the CPRQ and the physical and psychological wellbeing domain may be due to the small number of items used to measure them (two items for each). This is one of the key limitations of this study as outlined below.

Girls taking part in the KLT survey had higher scores on the CPRQ, and therefore more positive perceptions of their participation rights, than boys. In addition, the correlation between the social relations/autonomy domain and the CPRQ was stronger for girls than for boys. These findings support the results of previous research studies in relation to gender and participation in the contexts of family (González et al. 2015), school (Nelson et al. 2010) and in the community (Albanesi et al. 2015; Irvine and Osborne 2014; Wilson 2000). This gender difference in civic participation has been shown to persist into adulthood (Cicognani et al. 2012). Speculating on why this might be, González et al. (2015, p105) suggest that girls may be more socially responsible than boys and thus perceive participation ‘as an act of social responsibility’ and this is certainly a finding worth exploring in future research studies.

Perhaps the most informative finding to emerge from the present study is in relation to the questions on KIDSCREEN-10 that encompass children’s autonomy and relationships. Although KIDSCREEN-10 is a unidimensional measure, nonetheless, the questions assess wellbeing across three distinct domains – physical wellbeing, psychological wellbeing and social relations/autonomy. The findings from this study support the hypothesis proposed and indicate that the social relations/autonomy questions on KIDSCREEN-10 are most strongly related to children’s perceptions that their participation rights are respected in school and community contexts. This may shed some light on the nature of the relationship between child wellbeing and children’s participation rights. As discussed above, a rights-based approach to participation draws attention not only to the child’s autonomy but also to the role that adults play in supporting children in the exercise of their participation rights; for example, the extent to which children perceive that the adults in their lives not only listen to and respect their views but also make it easy for them to access information and to give their views. In short, it could be suggested that at the heart of the relationship between child wellbeing and children’s participation rights lies the social or relational aspects of both participation and wellbeing.

### Limitations of the Study

There are a number of limitations to the design of this study. Firstly, KLT is cross-sectional which means that it is not possible to determine cause and effect relations between wellbeing and participation rights using this method. Secondly, KIDSCREEN-10 is a unidimensional measure of wellbeing and we have chosen to construct three distinct domains from it for the purposes of this paper. Future research could perhaps use the CPRQ along with measures of subjective wellbeing which focus more on the social relationships and autonomy in children’s lives than the KIDSCREEN-10, such as the KIDSCREEN-27 or the Good Childhood Index (Rees et al. 2010), to further explore whether it is indeed this aspect of wellbeing that explains the relationship between child wellbeing and children’s participation rights.

### Conclusion

This paper has sought to add to the empirical evidence base and, importantly, to the emerging theoretical debate surrounding the relationship between children’s rights, in particular their participation rights, and their wellbeing. Whilst the findings from the study contribute to this discussion, it is important to sound a cautionary note. The evidence presented in this paper, and from the other studies involving knowledge of the CRC and participation, has been correlational. Intuitively, it may make sense to suggest that making children aware of their rights and enabling them to participate in decisions that affect them would lead to better subjective wellbeing. Indeed, Matthews (2003) notes that most cases for young people’s participation involve the claim that it enhances quality of life and encourages psycho-social wellbeing. However, it could be that children who have higher wellbeing are more likely to believe that their rights are respected or that other, as yet unmeasured, variables are mediating the relationship between wellbeing and children’s rights. This is something that cannot be answered at this time given the cross-sectional nature of the KLT survey. Future research, using a longitudinal design, would help to disentangle the causal nature of the relationship.

Based on the findings emerging from this study, and cognisant of the inherent limitations identified above, we would suggest that in order to understand the relationship between children’s participation rights and wellbeing, the nature and quality of the relationships and interactions between children and adults warrants further investigation. As Fattore et al. (2009, p61) suggest, children appear to understand wellbeing through the medium of their ‘significant relationships and emotional life’. Moreover they suggest that, from a child’s perspective, wellbeing relates not only to broader structural issues affecting their lives, but also to ‘small acts in daily interactions’ (p75). It would appear that in these interactions children want to have a sense of agency, or the capacity to exert influence; this they see as related to their wellbeing. In particular, Fattore et al. (2009, p64) suggest that, for children, it is ‘important to their wellbeing to be involved in more formal decisions about their lives’.

Notably, this characterisation of children’s interaction with adults aligns with the children’s rights-based perspective on participation adopted in this study: children should be supported by adults in developing their views, and have those views sought actively, listened to and taken seriously. Thus, the social or relational aspects of participation, in particular the ways in which children’s perceptions of autonomy and adult support for their participation intersect, emerge as salient. It could be suggested therefore that the extent to which adults respect, and support the development of, children’s autonomy and agency, and the extent to which they see children as rights-holders underpins not only the nature of their enjoyment of their participation rights but also their sense of wellbeing.

## Footnotes

1 The 15 countries were: Austria, Belgium, Bulgaria, Germany, Greenland, Luxembourg, Portugal, Romania, Russian Federation, Slovenia, Spain, Switzerland, Turkey, Macedonia and United Kingdom.

2 By convention, a Cohen’s d of .2 represents a small effect size; .5 represents a moderate effect size and .8 represents a large effect size.
